# Outcomes of primary anal sphincter repair after obstetric injury and evaluation of a novel three-choice assessment

**DOI:** 10.1007/s10151-018-1770-9

**Published:** 2018-03-15

**Authors:** K. Kuismanen, K. Nieminen, K. Karjalainen, K. Lehto, J. Uotila

**Affiliations:** 1 Department of Obstetrics and Gynecology, Tampere University Hospital, Tampere, Finland; 2 Faculty of Medicine and Life Sciences, University of Tampere, Tampere, Finland; 3 National Institute for Health and Welfare, Helsinki, Finland; 4 Department of Gastroenterology and Alimentary Tract Surgery, Tampere University Hospital, Tampere, Finland

**Keywords:** Anal sphincter, Anal incontinence, Fecal incontinence, Obstetric anal sphincter injury

## Abstract

**Background:**

The aim of the present study was to evaluate the subjective outcome of primary repair of obstetric anal sphincter injury (OASIS) at 6 months, the factors associated with the symptoms of anal incontinence (AI), and the role of a simple survey consisting in one question with three answer choices, combined with the Wexner incontinence score for the assessment of this patient population.

**Methods:**

A retrospective cohort study was conducted on patients with third- or fourth-degree OASIS operated on between January 2007 and December 2013 inclusive at Tampere University Hospital, Finland. At 6 months, the patients were asked to report their Wexner’s score as well as the three-choice assessment regarding AI symptoms. Based on this assessment, the patients were divided into three groups: those, asymptomatic, those with mild symptoms who did not want further treatment and those with severe symptoms who were willing to undergo further evaluation and treatment.

**Results:**

There were 325 patients (median age 30 years). A total of 310 patients answered the questionnaire. Of which, one hundred and ninety-eight (63.9%) patients were asymptomatic, 85 (27.4%) had mild AI, and 27 (8.7%) experienced severe symptoms. There was no statistical difference in the results between the two techniques used (overlapping vs. end-to-end), or the stage of specialization of the operating physician. Persistent symptoms were associated with instrumental vaginal delivery (OR 2.12, 95% CI 1.32–3.41), severity of the injury (OR 1.64, 95% CI 1.20–2.25), and increased maternal age (OR 1.07, 95% CI 1.02–1.13). The correlation between the three-choice symptom evaluation and the Wexner score was good (Spearman’s rho 0.82).

**Conclusions:**

After 6 months, severe symptoms after OASIS repair were present in 9% of women and were more frequent in older women, women with high-degree tears and after instrumental vaginal delivery. A three-choice assessment of AI symptoms correlated well with the Wexner score and might be useful to triage patients who need further evaluation.

## Introduction

Anal incontinence (AI) is defined by involuntary loss of feces or flatus [1]. The most common traumatic cause for AI in women is obstetric anal sphincter injury (OASIS) [2]. The incidence of obstetric third- and fourth-degree anal sphincter rupture varies from approximately 11% [3] worldwide to 0.6–4.2% in Nordic countries [4]. Known risk factors for OASIS are high fetal birth weight, long duration of second stage of delivery, operative delivery, primiparity, and midline episiotomy [5, 6].

AI despite primary OASIS repair has been reported to occur in 61% of patients [7]. The extent of sphincter damage [8], operative vaginal delivery [9], older age, and high body mass index (BMI) [10] are associated with the risk of fecal incontinence after primary repair.

In Finland, extensive and continuous efforts have been made to prevent OASIS and to improve the quality of diagnostics and repair [11].There has also been discussion about the optimal specialization (gynecologist or colorectal surgeon) of the operating physician and surgical team [12]. Outcomes after OASIS have improved with time, and the symptoms of anal incontinence are reported less frequently than previously [7].

The aim of this study was to examine the subjective outcome of OASIS primary repair surgery and to recognize the factors associated with persistent AI symptoms. We also evaluated the role of a simple three-choice assessment combined with the Wexner incontinence score [13] in patients with OASIS.

## Materials and methods

**A** retrospective cohort study on women with OASIS was conducted between January 2007 and December 2013 inclusive at Tampere University Hospital, Finland, a tertiary care teaching hospital with up to 5400 deliveries per year. The yearly cesarean section rate varied between 14.6% (2012) and 17.7% (2007), and the rate of operative vaginal deliveries from 5.6% (2013) to 8.1% (2008). At our hospital, the perineum is supported to prevent perineal tears in almost all deliveries [11]. According to the classification of OASIS, a third-degree injury involves the anal sphincters; (3a) involves less than 50% of thickness of the external anal sphincter, (3b) more than 50% of thickness of the external anal sphincter, and (3c) both the external and internal anal sphincter. Fourth-degree injury extends to the anorectal mucosa [14]. Our initial analysis was made of patients diagnosed with all types of third-degree injuries as well as fourth-degree obstetric anal sphincter injuries. Recurrent anal sphincter injuries (three cases, recurrence rate 5% of attempted vaginal deliveries) were excluded from the analysis.

At 6 months, the patients were sent a questionnaire about subjective AI symptoms including a Wexner incontinence score sheet and a three-choice assessment form. The Wexner incontinence score contains questions about the frequency and type of incontinence or discomfort (solid stool, liquid stool, flatulence; the use of diapers or pads, lifestyle changes). A score of 0 corresponds with full continence, while a score of 20 corresponds with total incontinence [13]. The empirically developed assessment asking patients which of three answer choices best described their condition is: (1) the patient is satisfied and has no symptoms (= no symptoms); (2) has mild symptoms but does not wish for a doctor’s appointment (= mild symptoms); and (3) has severe symptoms and wants to be contacted by a colorectal surgeon (= severe symptoms). This has been used to target the resources for women who want further treatment. The patients who reported severe symptoms were examined by a colorectal surgeon with endoanal ultrasound and anal manometry. A follow-up visit was arranged within 7–14 months of the initial injury.

**Fig. 1:**
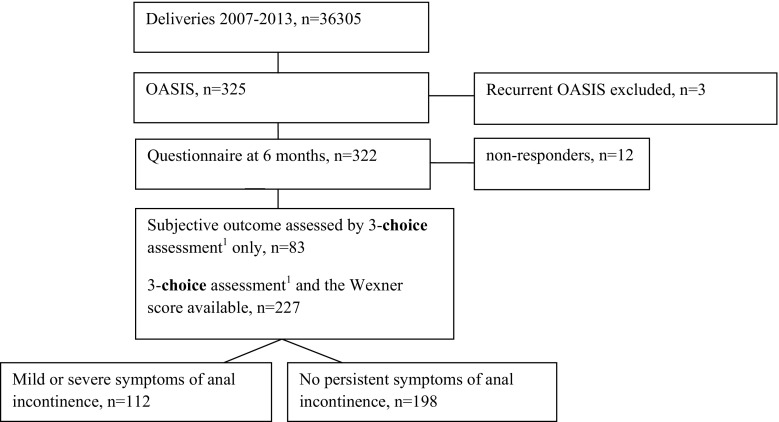
The patient flow chart

In order to examine the variables associated with persistent AI among patients diagnosed with third- and fourth-degree obstetric anal injuries, the women were divided into two groups based on the three-choice assessment: those who reported no AI symptoms at 6 months after OASIS (*n* = 198), and those who had minor or severe symptoms (*n* = 112). The study population is described in Fig. 1. The groups were compared using pre-labor and intrapartum factors.

### Statistical analysis

Statistical significance was tested by using Pearson’s Chi-square test with categorical and one-way analysis of variance (ANOVA) with continuous variables. Spearman’s rank correlation test was used to analyze the correlation between the Wexner incontinence score and the subjective outcome measure. The associations between different explanatory variables (Table 1) and the severity of symptoms were estimated by a logistic regression model, and the results were presented as odds ratios (OR) with 95% confidence intervals (CI). To distinguish between the asymptomatic and symptomatic groups, the severity of the anal incontinence symptoms (0 = no symptoms, 1 = mild/severe symptoms) was used as an outcome variable. In addition to univariate analysis, the models were adjusted for clinically valid variables, such as age, BMI, parity, and mode of delivery. A *p* value of < 0.05 was considered statistically significant. The data analysis was performed using IBM SPSS 22 software (Chicago, IL, USA).

**Table 1:** The demographic and clinical characteristics of the study groups

	No symptoms *n* = 198	Mild or severe symptoms *n* = 85 + 27 = 112	*p*	Univariate model OR (95% CI)	Multivariate model^a^ OR (95% CI)
Age (years)	29.0 (SD 4.5)	30.5 (SD 4.9)	0.015		
Age > 30	76 (38.4%)	59 (52.7%)	**0.007**	**1.79 (1.12**–**2.86)**	**1.75 (1.07**–**2.86)**
BMI (kg/m^2^)	24.1 (SD 4.6)	23.8 (SD 3.9)	0.456		
BMI > 30	21 (10.6%)	8 (7.1%)	0.314	0.65 (0.28–1.52)	0.67 (0.28–1.60)
Induction of labor	37 (18.7%)	28 (25.0%)	0.190	1.45 (0.83–2.53)	1.36 (0.76–2.43)
Oxytocin for augmentation	162 (81.8%)	94 (83.8%)	0.638	1.16 (0.62–2.16)	0.90 (0.47–1.73)
Duration of 2 stage					
≤ 5 min	6 (3.0%)	5 (4.5%)	0.017	1.97 (0.58–6.73)	2.06 (0.55–7.72)
5.01–44.99 min (ref)	130 (65.7%)	55 (49.1%)		1	1
≥ 45 min	62 (31.3%)	52 (46.4%)		**1.98 (1.22**–**3.22)**	1.41 (0.82–2.42)
Episiotomy	107 (54.0%)	73 (65.2%)	0.056	1.59 (0.99–2.57)	1.23 (0.71–2.23)
Epidural analgesia	138 (69.7%)	77 (68.8%)	0.862	0.96 (0.58–1.58)	0.81 (0.48–1.39)
Spinal analgesia	17 (8.6%)	8 (7.1%)	0.654	0.82 (0.34–1.96)	0.77 (0.30–1.98)
Birthweight (grams)	3742 (SD 493)	3704 (SD 480)	0.515		
weight > 4 kg	50 (25.3%)	31 (27.7%)	0.640	1.13 (0.67–1.91)	1.23 (0.71–2.14)
Head circumference mean cm	35.6 (SD 1.4)	35.3 (SD 1.5)	0.117		
Occipitoposterior position	21 (10.6%)	21 (18.8%)	**0.044**	**1.95 (1.01**–**3.75)**	1.81 (0.91–3.61)
Instrumental delivery	65 (32.8%)	57 (50.9%)	**0.002**	**2.12 (1.32**–**3.41)**	**2.06 (1.26**–**3.36)**
Classification of injury					
3a (ref)	100 (50.5%)	39 (34.8%)	**0.008**	**1.91 (1.18**–**3.08)**	**1.92 (1.17**–**3.15)**
3b/c/4	98 (49.5%)	73 (65.2%)			
Operation technique end-to-end (ref overlapping)	123 (62.1%)	58 (51.8%)	0.076	0.66 (0.41–1.05)	0.86^b^ (0.50–1.50)
Operated by a resident alone (ref specialist)	42 (21.2%)	17 (15.2%)	0.194	0.67 (0.36–1.23)	0.69 (0.36–1.30)

Statistically significant* p* values are shown in bold

Data presented as mean (SD) or *n* (%)

^a^Adjusted for maternal age, BMI, parity, mode of delivery

^b^Also adjusted for classification of injury

## Results

Three hundred and twenty-five patients were diagnosed with OASIS between the start of 2007 and the end of 2013 from a total of 36,305 deliveries. Primiparas accounted for 42.4% of in the total number of obstetric patients, but 76.6% of those who had OASIS. One hundred and ninety-four patients with OASIS (60.2%) had a spontaneous vaginal delivery (1 breech presentation), whereas 128 (39.8%) had an instrumentally assisted delivery (vacuum: 127, forceps: 1). A mediolateral episiotomy was performed in 188 (58.4%) cases), which differs from the total annual episiotomy rate in the hospital of 20–26% during the study period. The annual incidence of OASIS ranged from 0.69 to 1.10%.

All patients were operated on within 24 h of the delivery by either a resident, specialized gynecologist, or a colorectal surgeon. During their hospital stay, the patients received prophylactic antibiotics and had physiotherapy counseling including perineal inspection, pelvic floor exercise instructions as well as dietary information. The physiotherapist examined the patients and their pelvic floor function 3 months after the delivery.

Most of the patients had stage 3a (44.8%) or 3b (41.6%) tears, 11.6% had 3c, and only six patients (1.9%) had a stage 4 rupture. Sixty-five doctors were involved in the operations either operating or assisting, and the operation volumes per doctor varied from 1 single assisted operation to 31 performed operations. The operative technique was overlapping for 134 (41.2%) and end-to end for 191 (58.8%) patients, according to the operating surgeon’s preference. The end-to-end technique was used mostly (92.6%) for stage 3a or 3b ruptures, and all of the stage 4 ruptures were treated using the overlapping technique.

### Anal incontinence symptoms

According to the three-choice assessment, 198 (63.9%) women reported no symptoms at 6 months after OASIS repair and did not wish for an appointment with a doctor. Mild symptoms without need for a visit were reported by 85 (27.4%) women. Severe symptoms were reported by 27 (8.7%) women.

As shown in Table 1 (univariate model), advanced maternal age, long duration of the second stage of the delivery, occipitoposterior presentation, instrumental delivery, severe type of injury, and hospital stay of over 4 days were all associated with poorer outcomes at 6 months. However, in the multivariate model, only instrumental vaginal delivery, severe injury, and advanced maternal age persisted in being associated factors. Neither the operative technique nor the experience or specialization of the operating physician (resident gynecologist, specialist in gynecology or colorectal surgeon) was associated with poor outcome. The milder 3a tears were more often managed by a resident (34.5% of all 3a tears), whereas all the grade 4 tears were operated on by a senior gynecologist. Only 26 (8.1%) of the anal sphincter ruptures were operated on by a colorectal surgeon. Three out of 27 (11%) patients with persistent severe symptoms had a second sphincter repair operation. One of the patient**s** regained anal continence after the operation, while two remained incontinent. Most of the remaining 24 patients received extended pelvic floor physiotherapy and were satisfied with the results.

### Wexner incontinence score

**Fig. 2:**
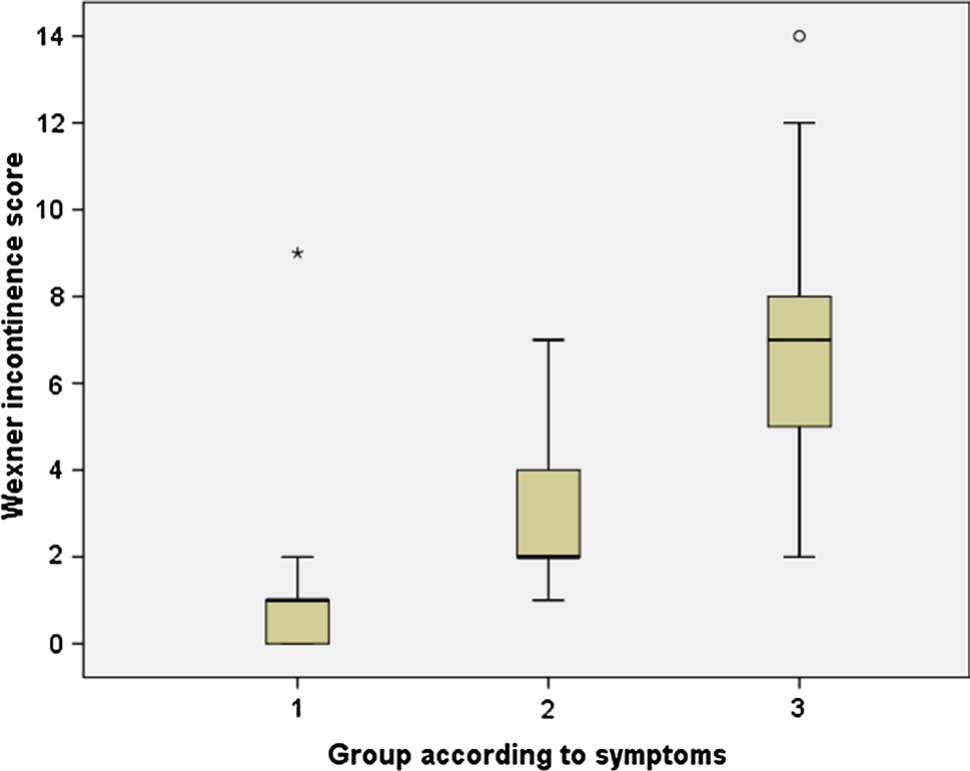
The correlation between the Wexner incontinence score and the three-choice assessment

The Wexner incontinence score was returned by 227 patients (70.1% of the total study population). The Wexner incontinence score was 0–4 for 193 (59.9%) patients, 5–6 for 17 (5.3%), and ≥ 7 for 17 (5.3%) patients. In the asymptomatic group, the Wexner score was 0–4 for 120 patients (99.2%), while 22 patients (81.5%) of those with severe symptoms had a Wexner score of 5 or higher. However, there was some discrepancy between the Wexner score and the patients’ perception of the severity of their symptoms; in the asymptomatic group, the Wexner incontinence score ranged from 0 to 9, and in the mildly or severely dissatisfied groups from 2 to 14. Nevertheless, the three-choice assessment and the Wexner incontinence score showed significant correlation (Spearman’s rho 0.82) (Fig. 2).

## Discussion

The overall short-term subjective results of OASIS primary repair were encouraging. However, 9% of the patients had severe symptoms at 6 months. This is similar to results of other studies, although other studies do not take into account subjective patient factors [15].

We found the grading of the injury to be an important prognostic factor. This finding supports that of Roos et al., where patients with grade 3c–4 injuries had a significantly poorer outcome than those with grade 3a–b injuries based on quality of life and the results of anal manometry. Women with major tears were also significantly more likely to have an internal and external anal sphincter defect detectable by endosonography [16].

Instrumental vaginal delivery was independently associated with AI at 6 months after primary sphincter repair. Johannessen et al. [17] discovered the occipitoposterior presentation to be the only prognostic factor for AI in primiparas. However, in our study, the association between the occipitoposterior position and AI disappeared after adjusting for maternal age, BMI, parity, and mode of delivery in multivariate logistic regression analysis. Neither was the duration of the second stage of delivery a prognostic factor for poor outcome. A mediolateral episiotomy has been considered as a protective factor from OASIS [18], but, in our study, the episiotomy did not seem to have an effect on AI symptoms after primary repair.

There was no statistical difference between the two repair techniques at 6 months. This is in contrast with some studies that found the overlapping technique was superior according to the symptoms at 12 months [19]. However, the milder ruptures were more often repaired using the end-to-end technique and the more severe ruptures with overlapping. The specialization or seniority of the surgeon did not seem to affect the results, although there was probably bias due to differences in assessment of the severity of the injury. The crucial step in OASIS treatment may be the recognition, and the operative technique seems to be less important.

The Wexner incontinence score is a simple and well established grading system [20]. The Wexner score of 9 has been considered a threshold for severe AI with significant impairment of the quality of life associate with scores higher than this [21]. Our study showed that patients with a Wexner score of 5 or higher reported severe symptoms. Therefore, a Wexner score of 4 might be a suitable threshold for further examinations to detect poor responders after primary repair in OASIS patients (young, previously healthy women). Additionally, in our study population, a simple three-choice assessment was very informative, as the correlation between the three-choice assessment and the Wexner score was good. The three-choice assessment takes the patient’s desire for further action into consideration and is informative especially when combined with the Wexner score. A visual analog scale (VAS) has also been used together with St Mark´s score to detect women who are troubled by AI [22]. Devesa et al. did not find sufficient agreement between the Wexner score and VAS and did not recommend the replacement of the validated AI scores [23]. In our opinion, the three-choice assessment might be a useful addition to current assessment methods.

The incidence of OASIS remained low during the study period, in contrast to some reports from Australia [24] and the UK [25]. The low incidence in Finland might be due to the practice of manually supporting the perineum when the baby’s head is crowning through the vaginal introitus [11]. In Israel, the incidence of OASIS has remained low, in spite of increased detection rate, due to the incorporation of manual perineal protection, the avoidance of midline episiotomy and the fact that the use of forceps is almost extinct [26]. In our study population, the recurrence rate of OASIS was 5%, which was similar to previous findings [27].

The long-term outcome of anal sphincter repair has to be further investigated. Farrell et al. found in their randomized trial comparing operative techniques that 39% (end-to-end) to 61% (overlapping) of primiparas suffered from flatus incontinence at 6 months, and at 3 years, flatus incontinence still bothered 39 and 43% of women, respectively [28]. In a Dutch cohort study, the women with OASIS had more than double the risk of long-term troublesome AI compared with the control group after a 4-year follow-up [29].

There are still women who need alternative treatment methods after OASIS primary repair. Secondary sphincteroplasty may not have the desired results, as the women often have denervated sphincters [30]. Sacral nerve modulation, as well as transcutaneous posterior tibial neuromodulation, has successfully been used for fecal incontinence, although follow-up times have been short [31]. Tissue engineering and stem cell therapy are future options and open new possibilities for the restoration of both the anatomy and function of the anal sphincter muscle [32–34].

This study reflects the usual practice in a large teaching hospital where over 60 doctors operated on OASIS patients during the study period. That outcome seems unrelated to specialty or experience deems not to support concentration of repair to the hands of a few.

Among the strengths of the study are the low drop-out volume, and the fact that a single center study enables thorough checking of every patient file to guarantee accurate classification of data. Our results reflect the patient’s own perception of the problem and the desire for further action, which might help in targeting health care resources. One limitation of the study is that not all patients returned the questionnaire and the Wexner score was missing in 27% of the cases. Other limitations are the retrospective nature of the study, as well as the lack of objective measurements (ultrasound, physical examination) of all the patients. Additionally, the three-choice assessment was not validated.

## Conclusions

Six months after delivery severe symptoms after OASIS repair were present in 9% of women. In addition to the Wexner score, a simple three-choice assessment of anal incontinence symptoms might be useful for evaluating the results of anal sphincter primary repair in OASIS patients and to survey the patient’s desire for further procedures.
